# Gingipain from *Porphyromonas gingivalis* causes insulin resistance by degrading insulin receptors through direct proteolytic effects

**DOI:** 10.1038/s41368-024-00313-z

**Published:** 2024-08-01

**Authors:** Fen Liu, Bofeng Zhu, Ying An, Zhifei Zhou, Peiying Xiong, Xuan Li, Yang Mi, Tongqiang He, Faming Chen, Buling Wu

**Affiliations:** 1 Shenzhen Stomatology Hospital (Pingshan), Southern Medical University, Shenzhen, China; 2 Key Laboratory of Shaanxi Province for Craniofacial Precision Medicine Research, Laboratory Center of Stomatology, Department of Paediatric Dentistry, College of Stomatology, Xi’an Jiaotong University, Xi’an, China; 3 State Key Laboratory of Oral & Maxillofacial Reconstruction and Regeneration, National Clinical Research Center for Oral Diseases, Shaanxi International Joint Research Center for Oral Diseases, Department of Periodontology, School of Stomatology, The Fourth Military Medical University, Xi’an, China; 4 Guangzhou Key Laboratory of Forensic Multi-Omics for Precision Identification, School of Forensic Medicine, Southern Medical University, Guangzhou, China; 5 Microbiome Medicine Center, Department of Laboratory Medicine, Zhujiang Hospital, Southern Medical University, Guangzhou, China; 6 Department of Stomatology, General Hospital of Tibetan Military Command, Lhasa, China; 7 Department of Stomatology Center, Shenzhen Hospital, Southern Medical University, Shenzhen, China; 8 Department of Obstetrics, Northwest Women’s and Children’s Hospital, Xi’an, China

**Keywords:** Bacterial pathogenesis, Diabetes, Preventive medicine

## Abstract

Periodontitis is a critical risk factor for the occurrence and development of diabetes. *Porphyromonas gingivalis* may participate in insulin resistance (IR) caused by periodontal inflammation, but the functional role and specific mechanisms of *P. gingivalis* in IR remain unclear. In the present study, clinical samples were analysed to determine the statistical correlation between *P. gingivalis* and IR occurrence. Through culturing of hepatocytes, myocytes, and adipocytes, and feeding mice *P. gingivalis* orally, the functional correlation between *P. gingivalis* and IR occurrence was further studied both in vitro and in vivo. Clinical data suggested that the amount of *P. gingivalis* isolated was correlated with the Homeostatic Model Assessment for IR score. In vitro studies suggested that coculture with *P. gingivalis* decreased glucose uptake and insulin receptor (INSR) protein expression in hepatocytes, myocytes, and adipocytes. Mice fed *P. gingivalis* tended to undergo IR. *P. gingivalis* was detectable in the liver, skeletal muscle, and adipose tissue of experimental mice. The distribution sites of gingipain coincided with the downregulation of INSR. Gingipain proteolysed the functional insulin-binding region of INSR. Coculture with *P. gingivalis* significantly decreased the INSR–insulin binding ability. Knocking out gingipain from *P. gingivalis* alleviated the negative effects of *P. gingivalis* on IR in vivo. Taken together, these findings indicate that distantly migrated *P. gingivalis* may directly proteolytically degrade INSR through gingipain, thereby leading to IR. The results provide a new strategy for preventing diabetes by targeting periodontal pathogens and provide new ideas for exploring novel mechanisms by which periodontal inflammation affects the systemic metabolic state.

## Introduction

Periodontitis is a chronic infectious disease caused by multiple periodontal pathogens. Without timely and effective treatment, periodontitis leads to continuous destruction of tooth-supporting tissues, which manifests as gingivitis, clinical attachment loss, alveolar bone resorption, bleeding on probing, and tooth loosening or loss.^1^ Forty-two percent of American adults have periodontitis, while 7.8% have severe periodontitis.^2^ Globally, approximately 11% of the population suffers from severe periodontitis, with 743 million people affected.^3^ Periodontitis not only affects mastication function but also has a great negative impact on the quality of life of patients.^4^

Periodontitis is a high-risk factor for a variety of systemic diseases. Among these diseases, diabetes is considered the most closely related to periodontitis. Patients with severe periodontitis usually have unfavourable blood glucose control and are more likely to have diabetes-related complications, such as cardiovascular disease, retinopathy, or diabetes neuropathy.^5^ Diabetes is a metabolic disease with high fatality and disability rates worldwide.^6^ By 2030, the number of diabetes patients worldwide may reach 552 million.^7^ Approximately 90% of diabetes patients suffer from type 2 diabetes, whose main pathological feature is ineffective insulin use by the body which leads to insulin resistance (IR). Local periodontal infections are believed to exacerbate IR.^8^ Compared with patients without periodontitis, type 2 diabetes patients with periodontitis usually show more severe IR, and effective periodontal treatment can reduce the severity of IR.^9^ Clinical studies based on the Asian population have also suggested that periodontal status plays an important role in the development of IR.^10^

The mechanism by which periodontitis affects IR has been a popular research topic in recent years. Early studies suggested that subgingival periodontal dysbacteriosis may lead to glucose intolerance in nondiabetic patients.^11^ In vivo studies have confirmed that periodontal pathogens can cause IR and glucose intolerance in mice.^12^ In addition, periodontal treatment aimed at controlling local bacterial infection improves the metabolic indexes of IR patients.^13^ These results suggest that periodontal pathogens may play key mediating roles in periodontitis and IR. Periodontal bacteria and their products may enter the blood circulation through damaged periodontal tissues,^14^ causing systemic inflammation, reaching insulin target organs and ultimately increasing the Homeostatic Model Assessment for IR (HOMA-IR) scores of periodontitis patients.^15^ However, several uncertainties remain to be addressed. First, there are numerous periodontal pathogens, and recent studies seldom have identified bacteria that play crucial roles. Second, the mechanisms by which the key periodontal pathogens cause IR after migrating to insulin target organs need to be further clarified.

In a previous population-based prospective clinical study, our team found that among the seven major periodontal pathogens, *Porphyromonas gingivalis* played the most important mediating role in periodontitis and gestational diabetes mellitus, the main pathological manifestation of which was IR.^16^ This discovery is also partially supported by a previous study in which changes in metabolic indicators in mice after subcutaneous injection of lipopolysaccharide from *P. gingivalis* were found to be similar to the effects of periodontitis on metabolism.^17^ Gingipain is one of the most important pathogenic factors of *P. gingivalis* and can invade and destroy host tissues through proteolytic activities.^18^ There are two types of gingipain, arginine- and lysine-specific. Both types can directly participate in the destruction of periodontal tissues and cause *P. gingivalis* to evade the host immune defence mechanism.^19^ As gingipain is a protease, its most significant biological function is to hydrolyse proteins. Insulin receptor (INSR) is a key protein in the process of insulin signal transduction.^20^ After binding with insulin, INSR transmits signals to the cell, initiating downstream pathways to ensure glucose transport.

Therefore, based on previous studies, this study aimed to explore the functional role and possible mechanisms of *P. gingivalis* in IR and tested the following hypothesis: *P. gingivalis* from the oral environment can migrate to insulin target organs, and in these organs, gingipain from *P. gingivalis* can exert a direct proteolytic effect to degrade INSR, affecting its binding to insulin and causing IR. The related results may provide a new strategy for preventing diabetes by targeting periodontal pathogens and new ideas for research on novel mechanisms of periodontal diseases that affect systemic metabolic status.

## Results

### Clinical isolation of *P. gingivalis* is significantly correlated with IR

The characteristics of the included participants were compared (Table 1). No significant differences were found in demographic, behavioural or health-related characteristics (*P* > 0.05). Except for clinical attachment loss (CAL), there was no difference in periodontal parameters between the groups. However, the incidence of periodontitis in the IR group was significantly greater than that in the control group (*P* = 0.01). In addition, the values of IR-related indicators (fasting glucose, fasting insulin and HOMA-IR) were greater in the case group than in the control group (*P* < 0.001). Then, the isolation rates of *P. gingivalis* 16 S rRNA in the saliva samples were analysed (Table 1). The results showed that the bacterial isolation rate in the IR group was 52.2%, which was greater than that in the control group (32.2%, *P* = 0.01). The crude relative risk (RR) was 2.30 (95% CI = 1.26–4.21). After controlling for confounding factors in the multiple logistic regression model (Table 2), we found that the adjusted odds ratio (OR) of positive isolation of *P. gingivalis* for IR was 2.25 (95% CI = 1.65–3.74), indicating that *P. gingivalis* may be a potential risk factor for IR.

**Table 1:** Comparison of the characteristics and isolation rates of *P. gingivalis* between the two groups

Characteristics	Control (*n* = 90)	Insulin resistance (*n* = 90)	*P* value
**Demographics**			
Age/year	43.1 ± 6.2	44.9 ± 6.3	0.06^a^
SES (%)			
SES-1	23(25.6)	21(23.3)	
SES-2	27(30.0)	26(28.9)	
SES-3	18(20.0)	23(25.6)	
SES-4	6(6.7)	13(14.4)	
SES-5	16(17.7)	7(7.8)	0.15^b^
Educational attainment (%)			
High school and below	52(57.8)	38(42.2)	
Undergraduate	26(28.9)	34(37.8)	
Graduate and above	12(13.3)	18(20.0)	0.11^b^
Sex (%)			
Male	33 (36.7)	44 (48.9)	
Female	57 (63.3)	46 (51.1)	0.10^b^
**Behaviours**			
Smoking (%)	8 (8.9)	16 (17.8)	0.08^b^
Alcohol consumption (%)	12 (13.3)	19 (21.1)	0.17^b^
**Health related**			
Body mass index	22.5 ± 3.6	23.6 ± 4.2	0.06^a^
Chronic hypertension (%)	3 (3.3)	6 (6.7)	0.31^b^
**Periodontal related**			
Diagnosis of periodontitis (%)	31 (34.4)	48 (53.3)	0.01^b^
Plaque index	1.9 ± 0.6	2.0 ± 0.4	0.19^a^
Sites with BOP/%	31.4 ± 12.9	33.3 ± 14.4	0.35^a^
PPD - Full mouth/mm	2.0 ± 0.6	2.1 ± 0.5	0.23^a^
CAL - Full mouth/mm	1.8 ± 0.5	2.0 ± 0.4	0.004^a^
**Insulin resistance related**			
Fasting glucose/ (mmol/L)	4.86 ± 0.58	7.88 ± 1.60	< 0.001^a^
Fasting insulin/ (µU/mL)	9.22 ± 2.39	13.64 ± 4.09	< 0.001^a^
HOMA-IR	1.97 ± 0.49	4.65 ± 1.29	< 0.001^a^
**Isolation of** ***P. gingivalis***			
Positive (%)	29 (32.2)	47 (52.2)	
Negative (%)	61 (67.8)	43 (47.8)	0.01^b,c^

*SES* socioeconomic status, *BOP* bleeding on probing, *PPD* probing pocket depth, *CAL* clinical attachment loss; *HOMA-IR* homeostatic model assessment for insulin resistance

^a^*t* test

^b^chi-squared test

^c^the crude relative risk of *P. gingivalis* positive isolation to negative isolation was 2.30 (95% CI = 1.26–4.21)

**Table 2:** Adjusted ORs of the risk of IR according to different characteristics determined by multiple logistic regression analysis

Characteristic^a^	B	SE	*P* value	Adjusted OR	95% Confidence interval	
					Lower	Upper
Age	0.65	0.27	0.14	1.85	0.46	2.99
Sex						
Male			1.00 (reference)			
Female	0.28	0.02	0.58	1.01	0.88	1.05
Smoking						
No			1.00 (reference)			
Yes	0.57	0.08	0.04	1.72	1.08	3.53
Body mass index	0.45	0.20	0.23	1.57	0.64	2.25
Diagnosis of periodontitis						
No			1.00 (reference)			
Yes	0.84	0.31	<0.001	2.42	1.49	3.48
CAL	0.38	0.13	0.06	1.52	0.93	2.42
Isolation of *P. gingivalis*						
No			1.00 (reference)			
Yes	0.76	0.14	<0.001	2.25	1.65	3.74

*OR* odds ratio, *IR* insulin resistance, *B* regression coefficient, *SE* standard error

^a^When determining one characteristic, the other characteristics in this regression model were used as controlled confounding factors

**Fig. 1:**
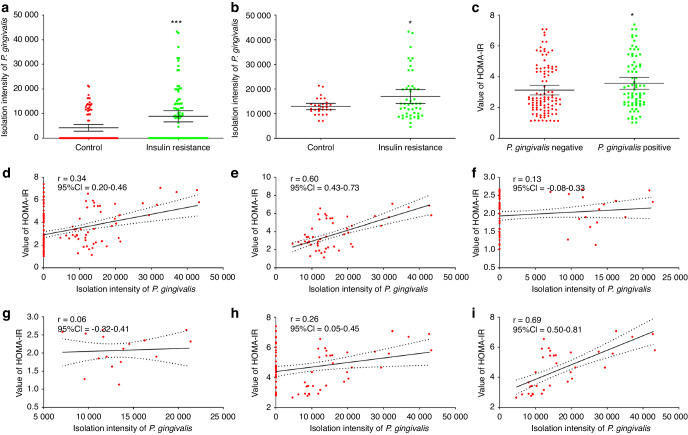
Clinical isolation of *P. gingivalis* is significantly correlated with IR. **a** Comparison of the amount of *P. gingivalis* isolated between the control and IR groups (mean and 95% CI). **b** Comparison of the amount of *P. gingivalis* isolated from *P. gingivalis*-positive samples between the control and IR groups (mean and 95% CI). **c** Comparison of HOMA-IR scores between *P. gingivalis*-positive and *P. gingivalis*-negative participants (mean and 95% CI). Correlation analysis between the amount of *P. gingivalis* isolated and the HOMA-IR score among the overall participants (**d**) and the *P. gingivalis*-positive participants (**e**). Correlation analysis between the amount of *P. gingivalis* isolated and the HOMA-IR score in the control group (**f**) and among *P. gingivalis*-positive participants in the control group (**g**). Correlation analysis between the amount of *P. gingivalis* isolated and the HOMA-IR score in the IR group (**h**) and among the *P. gingivalis*-positive participants in the IR group (**i**). IR, insulin resistance; *r*, correlation coefficient; CI, confidence interval; HOMA-IR: homeostatic model assessment for insulin resistance; *P < 0.05; ***P < 0.001

For all participants and *P. gingivalis*-positive participants, the average amount of isolated *P. gingivalis* was greater in the IR group than in the control group (Fig. 1a, b; *P* < 0.05). Further analysis suggested that the average HOMA-IR score of *P. gingivalis*-positive participants was greater than that of *P. gingivalis*-negative participants (Fig. 1c; *P* < 0.05). Correlation analysis suggested a positive correlation between the amount of isolated *P. gingivalis* and the HOMA-IR score in all participants (Fig. 1d; *r* = 0.34, *P* < 0.05). In *P. gingivalis*-positive participants, this correlation was further enhanced (Fig. 1e; *r* = 0.60, *P* < 0.05). In non-IR participants, there was no significant correlation between bacterial amount and HOMA-IR score (Fig. 1f, g; *P* > 0.05). In the IR population, there was a correlation between the amount of *P. gingivalis* isolated and the HOMA-IR score (Fig. 1h, i; *P* < 0.05), with the strongest correlation observed in IR participants with positive isolation of *P. gingivalis* (Fig. 1i; *r* = 0.69, *P* < 0.05). These results suggest that there is a significant correlation between the clinical isolation of *P. gingivalis* in saliva and the severity of IR.

### *P. gingivalis* may have functional effects on the occurrence of IR

**Fig. 2:**
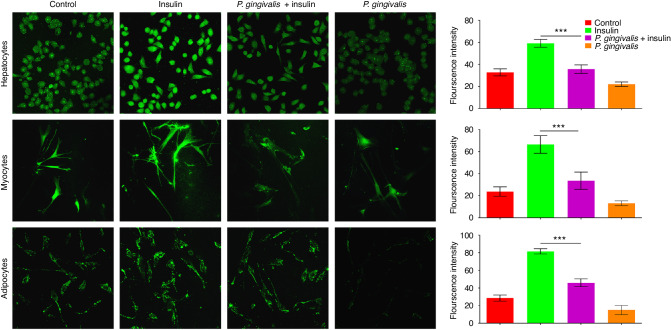
*P. gingivalis* may have functional effects on IR in vitro. Laser confocal microscopic observation of the changes in glucose uptake ability in hepatocytes (× 400), myocytes (× 200), and adipocytes (× 200) cocultured with *P. gingivalis* and the results of semiquantitative analysis. Green represents glucose that was taken up by the cells. ***P < 0.001

These previous results suggested a significant correlation between *P. gingivalis* and IR. The next experiment further explored whether this statistical correlation had clinical functional significance. For in vitro experiments, changes in the glucose uptake ability indicated differences among the three cell lines treated with insulin with or without *P. gingivalis*. Laser confocal microscopy suggested that compared with those in the normal control group, the glucose uptake abilities of hepatocytes, myocytes and adipocytes, which are all derived from the insulin targets and main organs involved in glucose metabolism, were greater in the insulin-treated group (Fig. 2). However, the promoting effects of insulin on the three kinds of cells were weakened after the cells were cocultured with *P. gingivalis* at a multiplicity of infection (MOI) of 100 (*P* < 0.05).

**Fig. 3:**
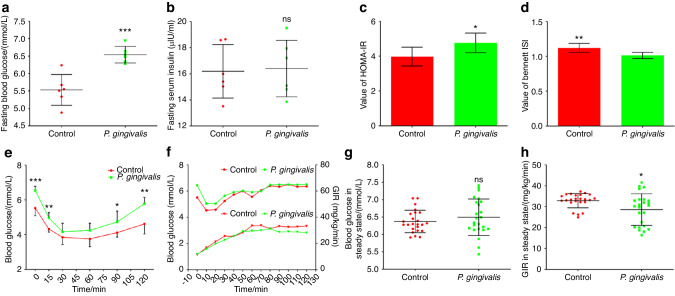
*P. gingivalis* may have functional effects on IR in vivo. Changes in fasting blood glucose (**a**) and fasting insulin (**b**) levels in mice orally administered *P. gingivalis* for 12 weeks (*n* = 6 in each group). Calculation of HOMA-IR (**c**) and ISI (**d**) based on fasting blood glucose and insulin measurements (*n* = 6 in each group). **e** Insulin tolerance test of mice orally administered *P. gingivalis* for 12 weeks (*n* = 6 at each time point). After oral feeding of *P. gingivalis* for 12 weeks, hyperinsulinaemic–euglycaemic clamp was performed to detect blood glucose levels (upper) and GIR (lower) at different time points in mice (**f,** *n* = 6 at each time point), and the differences in blood glucose levels (**g**) and GIR (**h**) in the steady state were compared. ISI insulin sensitive index, GIR glucose infusion rate, *P < 0.05; **P < 0.01; ***P < 0.001; ns, no significance

In vivo experiments showed that after oral administration of *P. gingivalis* for 12 weeks, the fasting blood glucose levels of the mice in the experimental group increased (Fig. 3a; *P* < 0.05), while the fasting insulin levels did not significantly differ between the groups (Fig. 3b; *P* > 0.05). For the mice in the experimental group, the HOMA-IR score was significantly increased (Fig. 3c; *P* < 0.05), while insulin sensitivity index (ISI) was decreased (Fig. 3d; *P* < 0.05). The insulin tolerance test (ITT) results indicated that 30 min after insulin injection, the blood glucose levels in both groups of mice tended to stabilize (Fig. 3e). At 60 min after insulin injection, the blood glucose levels of the mice in both groups showed an increasing tendency. Blood glucose in mice orally fed *P. gingivalis* increased faster at 90 and 120 min after insulin injection than in control mice (Fig. 3e; *P* < 0.05), indicating that the mice in the experimental group were less sensitive to insulin than the mice in the control group. Finally, a hyperinsulinaemic-euglycaemic clamp experiment was performed (Fig. 3f–h). After 80 min of clamping, the blood glucose levels of mice in both groups reached steady states (Fig. 3f). The difference in steady-state blood glucose levels between the two groups was not statistically significant (Fig. 3g; *P* > 0.05). However, the glucose infusion rate (GIR) was lower in mice that were orally administered *P. gingivalis* to maintain a stable blood glucose level (Fig. 3h; *P* < 0.05). The results of the clamp experiment indicated that after oral administration of *P. gingivalis* for 12 weeks, the mice tended to experience IR.

### Gingipain derived from *P. gingivalis* may degrade INSR

**Fig. 4:**
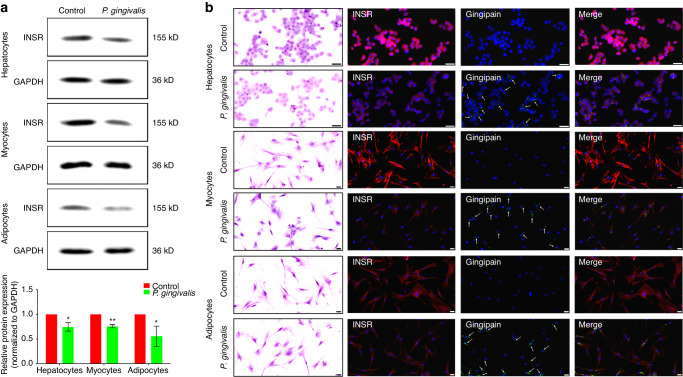
Gingipain derived from *P. gingivalis* may degrade INSR in vitro. **a** Western blot analysis of changes in INSR protein expression in hepatocytes, myocytes, and adipocytes after coculture with 100 MOI *P. gingivalis* and semiquantitative analysis (the expression intensity of proteins in the control group was set as 1). **b** Immunofluorescence staining was used to detect changes in INSR protein expression (red) in hepatocytes, myocytes, and adipocytes after coculture with *P. gingivalis* and to determine the distribution of gingipain (green, indicated by white arrows) (scale bar: 50 µm). INSR, insulin receptor; *P < 0.05; **P < 0.01

Previous results suggested that oral feeding of *P. gingivalis* may cause IR. The next experiment aimed to explore the possible target of *P. gingivalis* in inducing IR. Western blotting revealed that after coculture with *P. gingivalis* for 4 h, the protein expression of INSR in hepatocytes, myocytes, and adipocytes was significantly decreased (Fig. 4a; *P* < 0.05). In addition, immunofluorescence staining revealed that the sites of positive attachment of gingipain in the three types of cells overlapped with the sites of downregulated INSR (Fig. 4b; Supplementary Figs. 1–3 for magnified views).

**Fig. 5:**
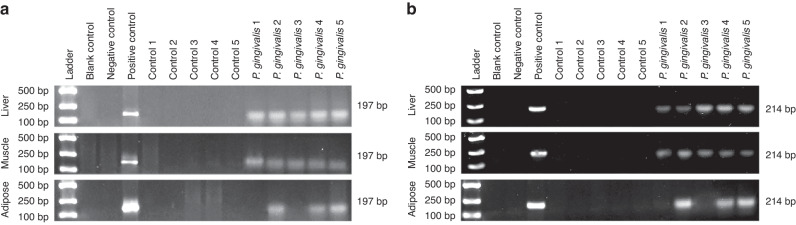
*P. gingivalis* may distantly migrated to insulin target organs. **a, b** PCR detection of the expression of *P. gingivalis* 16S rRNA (**a**) and the *P. gingivalis*-specific gene *hmuY* (**b**) in insulin target organs of mice

**Fig. 6:**
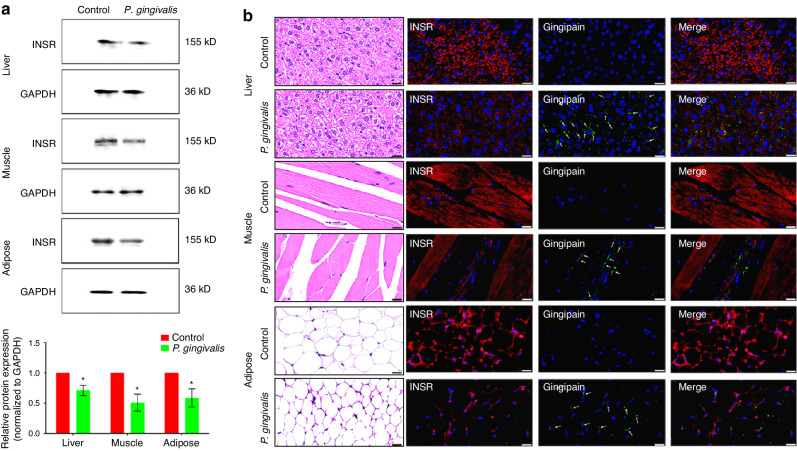
Gingipain derived from *P. gingivalis* may degrade INSR in vivo. **a** Western blot analysis of changes in INSR protein expression and semiquantitative analysis of INSR protein expression in insulin target organs of mice (the expression intensity of proteins in the control group was set as 1). **b** Immunofluorescence staining was used to detect changes in INSR protein expression (red) in insulin target organs of mice and the distribution of gingipain (green, indicated by white arrows) (scale bar: 20 µm). *P < 0.05

Five mice each were randomly selected from the control group and the experimental group to test for the presence of *P. gingivalis* in insulin target organs. Polymerase chain reaction (PCR) revealed no expression of *P. gingivalis* 16 S rRNA in the liver, skeletal muscle or adipose tissues of the mice in the control group (Fig. 5a), while *P. gingivalis* 16 S rRNA was detected in the liver and skeletal muscle tissues of 5 mice orally fed *P. gingivalis* and in the adipose tissues of 3 out of 5 mice in the experimental group (Fig. 5a). Further measurement of the expression of the *P. gingivalis*-specific gene *hmuY* showed similar results (Fig. 5b). PCR suggested that *P. gingivalis* fed orally could migrate to insulin target organs. The western blot results showed that after 12 weeks of oral feeding of *P. gingivalis*, the protein expression of INSR in the insulin target organs was downregulated (Fig. 6a; *P* < 0.05). Further immunofluorescence staining showed that gingipain was present in all three organs of mice in the experimental group and that the attachment sites of gingipain were basically consistent with the sites of downregulated INSR (Fig. 6b; Supplementary Figs. 4–6 for magnified views). The above results suggest that gingipain derived from *P. gingivalis* may cause IR by degrading INSR.

### Gingipain can directly hydrolyse the INSR α subunit and affect its binding to insulin

**Fig. 7:**
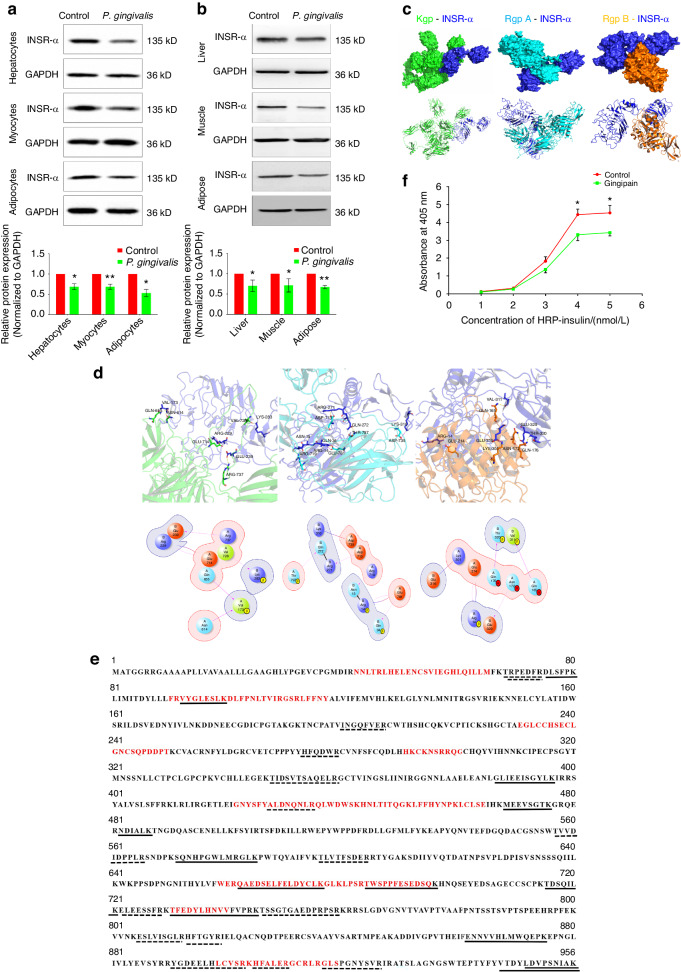
Gingipain can directly hydrolyse the INSR α subunit and affect its binding to insulin. **a** Changes in the protein expression of INSR in hepatocytes, myocytes and adipocytes after coculture with *P. gingivalis* and semiquantitative analysis of INSR (the expression intensity of proteins in the control group was set as 1). **b** Changes in the protein expression of the INSR α subunit in liver, skeletal muscle and adipose tissues after coculture with *P. gingivalis* and semiquantitative analysis of the INSR α subunit (the expression intensity of proteins in the control group was set as 1). **c** Molecular docking analysis between INSR and gingipain. Upper: surface pattern diagram; lower: ribbon pattern diagram. **d** Molecular docking analysis of the interaction between INSR and gingipain. Upper: 3D pattern diagram; lower: 2D pattern diagram. **e** LC‒MS/MS analysis of hydrolysis fragments of INSR after Kgp (solid lines) or RgpA/B (dotted lines) incubation; red amino acids indicated functional binding region of INSR. **f** ELISA of INSR–insulin binding ability changes after the effect of gingipain. INSR-α, insulin receptor α subunit; HRP, horseradish peroxidase; *P < 0.05; **P < 0.01

After coculture with *P. gingivalis*, the differences in the gene expression of INSR and INSR-A in hepatocytes, myocytes, and adipocytes were not statistically significant (Supplementary Fig. 7; *P* > 0.05). Similarly, after oral administration of *P. gingivalis* for 12 weeks, the mRNA expression of INSR or INSR-A in the insulin target organs of the mice did not significantly change (Supplementary Fig. 7; *P* > 0.05). The western blot results showed that the expression of the INSR α subunit was significantly downregulated both in vitro and in vivo (Fig. 7a, b; *P* < 0.05), indicating that the effects of *P. gingivalis* on INSR were mainly at the post-transcription.

The results of the molecular docking analysis suggested that there were direct binding sites between the lysine-specific gingipain (Kgp), the arginine-specific gingipains (Rgps: RgpA and RgpB) and INSR (Fig. 7c), mainly hydrogen bonds and salt bridges (Fig. 7d). The molecular docking results were further verified by liquid chromatography (LC)-mass spectrometry (MS)/MS (Fig. 7e). After 30 min of coculture with Kgp, 13 hydrolysed fragments of the INSR α subunit were detected, of which 4 were located in the functional regions where INSR binds to insulin. In contrast, after coculture with RgpA/B, 15 hydrolysed fragments were detected, of which 4 were located in the functional regions. The results of LC-MS/MS indicated that both Kgp and Rgp of gingipain may affect the binding of insulin to INSR through direct hydrolysis. The enzyme-linked immunosorbent assay (ELISA) results further supported this hypothesis (Fig. 7f). After coculture with *P. gingivalis*, INSR-insulin binding significantly decreased with increasing insulin concentrations (*P* < 0.05).

### Gingipain knockout alleviates IR caused by *P. gingivalis*

**Fig. 8:**
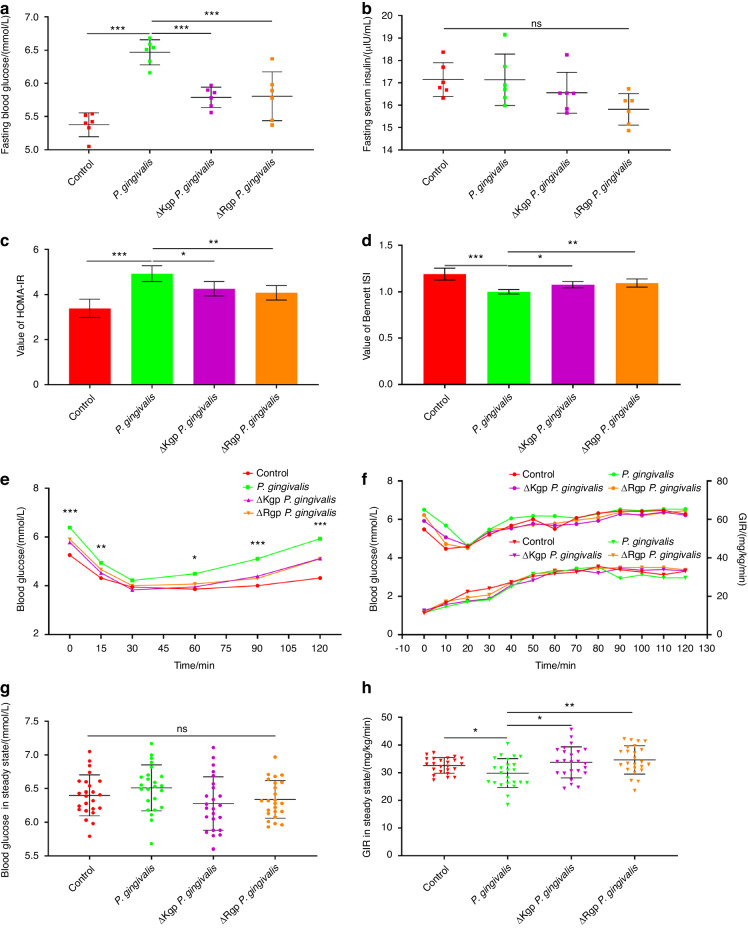
Gingipain knockout alleviates IR caused by *P. gingivalis*. Changes in fasting blood glucose (**a**) and fasting insulin (**b**) levels in mice orally administered *P. gingivalis* for 12 weeks (*n* = 6 in each group). Calculation of the HOMA-IR score (**c**) and ISI (**d**) based on fasting blood glucose and insulin measurements (*n* = 6 in each group). **e** Insulin tolerance test of mice orally administered *P. gingivalis* for 12 weeks (*n* = 6 at each time point). After oral feeding of *P. gingivalis* for 12 weeks, hyperinsulinaemic–euglycaemic clamping was performed, and the blood glucose levels (upper) and GIR (lower) were measured at different time points in the mice (**f,** *n* = 6 at each time point). The differences in blood glucose levels (**g**) and GIR (**h**) in the steady state were compared. *P < 0.05; **P < 0.01; ***P < 0.001; ns, no significance

Previous results suggested that the direct hydrolysis of INSR by gingipain may lead to IR. In this study, gingipain knockout *P. gingivalis* was used in vivo. Compared with that of wild-type *P. gingivalis*, oral administration of gingipain-knockout (Kgp or Rgp) *P. gingivalis* resulted in decreased fasting blood glucose levels in mice (Fig. 8a; *P* < 0.05). Although there was no difference in fasting insulin among the groups (Fig. 8b), after gingipain knockout, the HOMA-IR scores of the experimental mice were decreased (Fig. 8c; *P* < 0.05), while the ISI values were increased (Fig. 8d; *P* < 0.05). The ITT results also indicated that after Kgp or Rgp knockout, the sensitivity of the experimental mice orally fed *P. gingivalis* to insulin increased (Fig. 8e). The results of the hyperinsulinaemic–euglycaemic clamp experiment (Fig. 8f) indicated that when blood glucose reached a steady state (Fig. 8g), the GIR of the mice in the gingipain-knockout group was greater than that of the mice orally fed wild-type *P. gingivalis* (Fig. 8h; *P* < 0.05). These results suggest that gingipain knockout alleviates IR in mice oral feeding of *P. gingivalis*.

## Discussion

This study aimed to explore the functional role and possible mechanisms of *P. gingivalis* in IR. The clinical data suggested a significant correlation between the amount of *P. gingivalis* isolated from saliva and the severity of IR. In vitro and in vivo experiments further confirmed that this correlation has clinical functional value and that gingipain derived from *P. gingivalis* may cause IR by degrading INSR. Specifically, gingipain may directly enzymatically hydrolyse the INSR α subunit at the post-transcription to disrupt the binding of INSR to insulin.

In clinical work, the gold standard for diagnosing IR is hyperinsulinaemic–euglycaemic clamping.^21^ However, this is an invasive test that is expensive and time-consuming. Therefore, an increasing number of scholars are using HOMA-IR to quickly determine the presence of IR.^22^ According to the World Health Organization,^23^ a HOMA-IR value greater than the 75th percentile of the nondiabetic background population is considered diagnostic for IR. Therefore, we chose a value of 2.69, corresponding to the value of the 75th percentile in the study with the largest sample size (*n* = 10 147) carried out thus far in a population with normal glucose tolerance in China, as the cut-off point for diagnosing IR in this study.^24^ However, we also note that there are significant differences in the definition of the cut-off HOMA-IR score among different studies.^25–27^ How to determine the cut-off point affects the identification of IR, thus further affecting the health maintenance of populations with different sexes, ages, races, and disease backgrounds. More research is needed to determine the most accurate and targeted IR values in populations with different backgrounds.

Previous studies have suggested that the occurrence of diabetes is related to bacterial infection.^28^ Periodontal disease is an important risk factor that may lead to diabetes. The prevalence of type 2 diabetes in patients with severe periodontitis is significantly increased,^8^ mainly because of dysbacteriosis of periodontal pathogens in the inflammatory state.^29^ In vivo studies have also confirmed that exposure to periodontal pathogens can lead to IR and glucose intolerance in mice.^12^ Effectively treating periodontitis and controlling local bacterial infection can improve the metabolic indicators of patients with type 2 diabetes and improve the treatment effects of blood glucose control.^30^ The possible mechanism by which periodontal pathogens lead to IR is through interference of migrated bacteria or bacterial components with insulin signalling pathways. Increasing adoptive immune responses against periodontal pathogens such as *P. gingivalis* can alleviate glucose intolerance symptoms in mice after periodontal infection.^17^

This study focused on the association between *P. gingivalis* and IR. The results of the in vitro experiments suggested that after coculture with *P. gingivalis*, the glucose uptake ability of the cells was significantly reduced. In vivo experiments indicated that oral feeding of *P. gingivalis* led to glucose intolerance and IR in experimental mice. These results further confirm that *P. gingivalis* has a functional correlation with IR. These results were essentially consistent with those of previous studies. Previous in vivo experiments in mice have shown that *P. gingivalis* can exacerbate glucose intolerance and IR.^17^ Oral feeding of *P. gingivalis* can also cause IR and macrophage infiltration in the adipose tissues of the epididymis, upregulating the expression of multiple inflammatory cytokines and downregulating the gene expression of insulin sensitivity regulatory factors.^31^ In addition to adipose tissues, after oral feeding of *P. gingivalis*, the skeletal muscles of mice exhibit IR symptoms.^12^ Other researchers have shown that *P. gingivalis* can interact with type 2 diabetes-related genes, leading to changes in disease susceptibility.^32^ Thus, the current study not only confirms the findings of previous studies but also provides new references for in-depth mechanistic studies.

INSR plays important mediating roles in the function of insulin and the related signalling pathway transduction processes.^33^ INSR is a tetrameric protein consisting of two extracellular α subunits and two transmembrane β subunits. The four subunits are connected by disulfide bonds. The first step in the functioning of insulin is to connect to INSR on the surface of the target cell membrane, specifically to the extracellular portion of the INSR α subunit.^34^ Previous research has tended to focus on INSR after insulin binds to the α subunit and the changes in signalling pathways that are mediated by β subunit transduction and downstream INSR substrates in physiological or pathological states, such as the phosphorylated PI3K-akt signalling pathway.^35^ However, few studies have focused on the underlying mechanisms of changes in these signalling pathways or upstream protein level changes. The results of this study suggest that the migration of gingipain, an important toxic factor of *P. gingivalis*, can affect the binding of insulin to INSR through direct proteolytic degradation in insulin target organs, thus causing IR and glucose intolerance in skeletal muscle, liver and adipose tissues. These results supplement those of previous studies on signalling pathways and reveal the possible upstream destructive effects of *P. gingivalis* on glucose metabolic disorders.

To demonstrate that gingipain derived from *P. gingivalis* has a direct destructive effect on INSR in insulin target organs, we sought to confirm that *P. gingivalis* can migrate from the periodontal microenvironment to insulin target organs. We demonstrated this through end-point PCR and immunofluorescence staining. The finding that *P. gingivalis* can migrate to distant organs is consistent with the results of previous studies. Other scholars have detected periodontal pathogens, including *P. gingivalis*, in 73% of serum samples from Alzheimer’s disease patients and detected gingipain in brain tissues.^36^ In another animal study, the authors found that *P. gingivalis* can migrate to the umbilical cord through damaged periodontal tissues and cause adverse pregnancy outcomes such as low birth weight.^37^ In recent years, studies have also supported that the occurrence and development of vascular diseases are closely related to periodontal pathogens. Periodontal pathogens can escape host immune defences and reach and colonize atherosclerotic plaques and thrombi through the blood circulation. A variety of periodontal pathogens, including *P. gingivalis*, can be detected in these pathological tissues.^38^ After entering the circulatory system, *P. gingivalis* can also change the intestinal microbial composition of diabetic mice, reducing insulin sensitivity, increasing fasting blood glucose, and leading to IR in high-fat diet-fed mice through the branched-chain amino acid synthesis pathway.^39^ Our research, along with these findings, suggests the possibility of distant migration of periodontal pathogens. Maintaining periodontal health may thus be an important means of preventing systemic diseases.

The results of this study suggested that the direct destructive effect of *P. gingivalis* on INSR is attributable to the important virulence factor gingipain. Gingipain can destroy host proteins through its proteolytic effect so that *P. gingivalis* can obtain nutrients and exert toxic effects to cause a variety of systemic reactions.^40^ In fact, most of the proteolytic activity of *P. gingivalis* comes from gingipain. In recent years, studies have shown that the outer membrane vesicles (OMVs) of *P. gingivalis* are involved in bacterial destruction, and the most important substance inside the vesicles is also gingipain.^41^ Inhibiting the activities of gingipain can greatly reduce the haemolytic activity of *P. gingivalis*, thereby reducing its toxicity.^42^ This study revealed that the three gingipain virulence factors Kgp, RgpA, and RgpB all had hydrolytic effects on the INSR α subunit. Both Rgp and Kgp yielded hydrolytic fragments located in the functional regions of the INSR α subunit; thus, their binding with insulin was hindered. Some hydrolytic fragments of the INSR α subunit after incubation with Rgp or Kgp are located in the nonfunctional regions of the receptor. The nonfunctional regions of the INSR α subunit do not directly bind to insulin, but changes in their amino acid sequences or chemical modifications can significantly affect the affinity of the functional regions for insulin binding and regulate the activity of insulin bound to the receptors.^33^ Both Rgp and Kgp exert direct effects (hydrolysis of the functional regions of the INSR α subunit) and indirect effects (hydrolysis of the nonfunctional regions of the INSR α subunit) to affect the binding of insulin to the INSR. From this perspective, we believe that the cleavage profiles of Rgp and Kgp with the INSR α subunit are consistent. The results are consistent with those of previous studies, further increasing understanding of the pathological spectrum of gingipain damage.

There were some limitations in this study. First, this study focused on the direct hydrolysis of INSR by gingipain from *P. gingivalis*. However, bacteria also have other indirect effects on INSR and insulin signal transduction in target organs. This needs further exploration in later research. Second, *P. gingivalis* has many virulence factors. In addition to gingipain, there are also fimbriae, lipopolysaccharide, outer membrane proteins, haemagglutinin, *etc*.^43^ These virulence factors contribute significantly to the destructive effects of *P. gingivalis*. In addition to gingipain, other virulence factors need to be further evaluated to comprehensively characterize the toxic effects of *P. gingivalis*. Third, INSR is also expressed in other organs,^20,34^ such as the kidney, brain, and retina. The role these organs play in glucose metabolism is not as important as that of the liver, skeletal muscle or adipose tissue. However, these organs are also potential insulin target organs. In future studies, pathological functional changes associated with the effect of *P. gingivalis* should also be considered. Finally, the roles of the OMVs of *P. gingivalis* in transporting proteins, lipids, or nucleic acids have been popular research topics in recent years.^44^ The OMVs of *P. gingivalis* are smaller than the bacteria themselves, making it easier for the OMVs to evade host immune responses and penetrate deep tissues. This study focused on the distant migration of bacteria; whether *P. gingivalis* from the periodontal microenvironment can affect systemic diseases by releasing OMVs is a future research direction.

Through clinical research, in vitro cytological experiments, and in vivo animal experiments, we found that *P. gingivalis* in the oral cavity may migrate to insulin-target organs and degrade INSR through the direct proteolytic activity of gingipain (Kgp/Rgp). These pathological processes may lead to the deterioration of INSR–insulin binding and the occurrence of IR. The results provide a new strategy for preventing diabetes by targeting periodontal pathogens and provide new ideas for research on the novel mechanisms by which periodontal inflammatory disease affects systemic metabolic status.

## Materials and methods

### Clinical research design

All health screening examinees from the Department of Endocrinology in Northwest Women’s and Children’s Hospital from March to June 2022 were invited to participate in this study. The exclusion criteria for participants (in both the control and case groups) were ① those with tumours, liver diseases, asthma, autoimmune diseases, blood system diseases, chronic renal failure, and other systemic diseases; ② those with HIV infection and other infectious diseases; and ③ those who had used antibiotics or hormones within 3 m before participating. According to the diagnostic criteria for IR, the participants included were divided into healthy (control) and IR (case) groups with equal numbers. The included participants filled out structured questionnaires to collect basic information, and their venous blood and saliva were also collected for subsequent testing.

### Sample size calculation

In 2021, a cross-sectional pilot study was conducted at Northwest Women’s and Children’s Hospital. In this study, 100 healthy and 100 IR participants were randomly selected. These participants, who were between 32 and 56 years old, all signed informed written consent forms. The percentage of *P. gingivalis* isolated from the saliva of healthy participants was 31.0%, while that from the saliva of IR participants was 56.0%. Based on 90% power (*β* = 0.1) and a 5% significance level (α = 0.05) to detect differences between groups, PASS 2021 software (version 21.0.3, NCSS Statistical software, East Kaysville, Utah, USA) was used to calculate the needed sample size for this study. The results suggested that at least 80 participants were needed in each group. To account for an estimated 10% sample loss, 90 participants were ultimately included in each group.

### Structured questionnaires

All participants were distributed structured questionnaires to collect relevant information. The structured questionnaire was divided into three main parts: demographic characteristics, behaviours, and health-related information. The demographic characteristics included age, sex, educational attainment, occupation and annual family income. Based on educational attainment, occupation, and annual family income, socioeconomic status (SES) was calculated.^45^ According to the rating, the SES was divided into 5 levels. Behaviours included smoking and alcohol consumption. Participants who smoked more than 100 cigarettes per year for more than 1 year were considered to have a smoking habit. Participants who consumed more than 1 g of alcohol per day consistently within the past month were considered to have an alcohol consumption habit.^46^ Health-related information included body mass index (BMI) and history of hypertension. BMI was calculated as height (m) divided by weight (kg) squared. The Cronbach’s α coefficient was 0.91, indicating good credibility for this questionnaire. All questionnaires distributed in this study were collected, indicating good acceptability.

### Periodontal examination and diagnosis of periodontitis

The periodontal examination was performed by two trained and calibrated dentists using Williams periodontal probes (Hu-Friedy, Chicago, IL, USA). The clinical examination for supragingival dental plaque was conducted using the Silness and Löe plaque indices. The periodontal probing pocket depth (PPD), CAL, and bleeding on probing (BOP) in each tooth, except for the third molars, were examined at six sites. PPD was measured from the gingival margin to the bottom of the gingival sulcus or periodontal pocket. The CAL was determined from the cementoenamel junction to the deepest site of probing. Interrater and intrarater reliability were assessed in a random group of 20 patients with periodontitis. The kappa coefficient of the interrater agreement for PPD was 0.86, and that for CAL was 0.88. For intrarater agreement, the same patient was examined twice by one examiner at an interval of 1 week. Periodontitis was defined as an interdental CAL detectable in ≥ 2 nonadjacent teeth or a buccal or oral CAL ≥ 3 mm with a PPD ≥ 3 mm in ≥ 2 teeth.

### Diagnosis of IR

After fasting for at least 12 h, 5 ml of venous blood was collected from each participant. Via centrifugation at 3 000 × *g* for 15 min, the serum was separated. Fasting glucose was measured by an automatic biochemical analyser (Beckman AU5800, Indianapolis, Indiana, USA). The insulin concentration was tested using a chemical immunoluminescence kit (Elecsys Insulin, Roche Diagnostics GmbH, Mannheim, Germany). The HOMA-IR score was calculated as follows: fasting glucose (mmol/L) * fasting insulin (µU/mL)/22.5. In this study, the cut-off HOMA-IR score to diagnose IR was selected as 2.69.^24^

### End-point PCR

After fasting overnight, saliva not produced due to irritation was collected into a polypropylene tube. After centrifugation at 800 × *g* at room temperature for 10 min, 500 μL of supernatant was collected and immediately freeze-dried. Salivary DNA was extracted with a DNA extraction kit (DNA-EZ Reagents V All-DNA-Fast-Out, Ziker Biological Technology, Shenzhen, Guangdong, China). Briefly, 2 μL of saliva or 2 mg of tissue sample was added to 50 μL of reagent. After heating at 80 °C for 5 min, the lysate was taken for subsequent PCR analysis. DNA concentrations were determined using a NanoDrop 2000 (Thermo Fisher Scientific, Waltham, Massachusetts, USA).

The reverse transcription PCR system was 25 μL, containing 0.5 μL of DNA template (the DNA concentration was 20–50 µg/μL), 2.5 μL of 10× buffer, 1 μL of dNTPs (Sangon Biotechnology, Shanghai, China), 0.2 μL of DNA polymerase (Fermentas, Burlington, Ontario, Canada), 0.5 μL of forward and reverse primers (10 mol/L for each), and distilled water. A PCR thermocycler (Applied Biosystems, Carlsbad, California, USA) was used for the reaction. The reaction processes were as follows: predenaturation at 94 °C for 2 min; 35 cycles at 94 °C for 30 s, 55 °C for 30 s, and 72 °C for 30 s; an extension reaction at 72 °C for 10 min; and termination at 4 °C. Ten microlitres of amplification product was subjected to 1% agarose (BBI Life Sciences, Shanghai, China) gel electrophoresis. After being stained with ethidium bromide, the DNA was observed with a 300 nm ultraviolet transilluminator. Photoshop (version 2021, Adobe Systems, San Jose, California, USA) was used for semiquantitative analysis of the PCR products.

The blank control was a DNA template-free reaction system. The negative control contained DNA extracted from *Helicobacter pylori*, and the positive control contained DNA extracted from the *P. gingivalis* W83 strain. The forward primer used for *P. gingivalis* 16S rRNA was TGTAGATGACTGATGGTGAAAACC, while the reverse primer used was ACGTCATCCCCACCTTCCTC. The forward primer for the *hmuY* gene of *P. gingivalis* was ACCATAAACACACGGAATAATCG, while the reverse primer was GATATTGCCGGATACGATGG.

### Cell culture

Normal human liver (LO2) cells were purchased from the American Type Culture Collection (ATCC, Rockville, Maryland, USA). The LO2 cells were cultured in Dulbecco’s modified Eagle’s medium (DMEM) containing 10% foetal bovine serum, 100 U/mL penicillin, and 100 µg/mL streptomycin (Gibco, Carlsbad, California, USA) in a humid environment containing 5% CO_2_ and 95% air at 37 °C.

Human skeletal muscle cells and adipose tissue-derived cells were donated by Xi’an Tank Medicinal Biology Institute. Myocytes were cultured in low-glucose (1 g/L) DMEM containing 8% foetal bovine serum, 0.4 µg/mL dexamethasone (Sigma, Saint Louis, Missouri, USA), 10 ng/mL epidermal growth factor (Abcam, Cambridge, Cambridge, UK), 50 µg/mL foetal globulin (Sigma), 0.1% gentamicin (Gibco) and 0.1% amphotericin B (Gibco). Adipocytes were cultured in a specific culture medium (Cyagen, Santa Clara, California, US). All culture media were changed every 2-3 d.

### Growth of P. gingivalis

Culture of *P. gingivalis* was carried out according to previous studies.^19^ In brief, the W83 wild-type strain of *P. gingivalis* (ATCC) and the W83 strain-derived strains ΔKgp *P. gingivalis* and ΔRgp *P. gingivalis* (kindly provided by Prof. Yuan Gao from the College of Pharmacy, Fourth Military Medical University) were grown in brain–heart infusion fluid (Sigma) supplemented with 1 µg/mL vitamin K, 5 µg/mL sanguinin and 5% fibrin-free sheep blood in an anaerobic chamber at 37 °C. The growth environment contained 80% N_2_, 10% H_2_, and 10% CO_2_. The bacterial concentration was measured by a spectrophotometer at a wavelength of 600 nm.

### Glucose uptake assay

Cells were seeded into six-well plates and cultured with glucose-free DMEM. After 2 h of culture, the cells in the experimental group were cocultured with *P. gingivalis* at a MOI of 100 for 4 h. Afterwards, 100 nmol/L insulin (Yeasen, Shanghai, China) was added, and the cells were incubated for 1 h. To analyse glucose uptake ability, differently treated cells were incubated with 100 μg/ml 2-deoxy-2-[(7-nitro-2,1,3-benzoxadiazol-4-yl) amino]-D-glucose (Abcam) at 37 °C for 1 h. The fluorescence was observed by laser confocal microscopy. The fluorescence excitation wavelength was 485 nm, and the emission wavelength was 535 nm. Five different visual fields in each group were randomly selected. Integrated optical density measurements were obtained using Image-Pro Plus 6.0 software (Azure Biosystems, Dublin, CA, USA).

### Animals

Specific pathogen-free male C57BL/6 mice aged 6–8 weeks were purchased (Guoruiyinuo Co., Ltd., Xi’an, Shaanxi, China) for *P. gingivalis* oral feeding experiments. The mice weighed 20–25 g and were housed in independent ventilated cages with unrestricted access to a standard laboratory diet and drinking water. The mice were kept under a 12-hour light/dark cycle at a room temperature of (22 ± 2) °C and a relative humidity of (60 ± 5) %. The mice in the control and experimental groups were raised separately. All animal experiments were reviewed and approved by the Ethics Review Committee of Shenzhen Stomatological Hospital (Pingshan) of Southern Medical University (2022-407).

The results of preliminary mouse experiments (10 mice in each group) suggested that the fasting blood glucose level of the control group was (5.46 ± 0.57) mmol/L, while that of the group in which the mice were orally infected with *P. gingivalis* was (6.36 ± 0.75) mmol/L. Based on 90% power (β = 0.1) and a 5% significance level (α = 0.05) to detect differences between groups, PASS 2021 software was used to calculate the minimal number of mice needed. The results indicated that at least 6 mice were needed in each group (or at each time point).

### *P. gingivalis* oral infection

*P. gingivalis* oral infection experiments on mice were carried out as described in previous studies.^36^ Before the experiment, mice were given an intramuscular injection of 20 mg/kg ampicillin (Solarbio, Beijing, China) every day for 3 consecutive days. Before constructing the animal model, the mice were intraperitoneally injected with 25 mg/kg pentobarbital sodium (BioChemPartner, Shanghai, China). Afterwards, 5-0 silk threads (Johnson & Johnson, New Brunswick, New Jersey, USA) were tied around the maxillary second molars on both sides under the gingiva for mice in the control group and the experimental group. The mice in the experimental group were given 100 μL (1 × 10^10^/mL) of *P. gingivalis* W83 solution on the buccal sides of the maxilla every other day. The bacterial solution was phosphate-buffered saline (PBS, Servicebio, Wuhan, Hubei, China) containing 2% sodium hydroxymethyl cellulose (Coolaber, Beijing, China). The mice in the control group were given only PBS without *P. gingivalis*. After 12 w, IR-related indicators were measured. Finally, the mice were sacrificed, and the liver, lower limb gastrocnemius muscle and inguinal adipose tissues were taken for follow-up studies.

### Biochemical testing of mice

The cleaned tails of the mice were immersed in 50 °C water for 3 min. Afterwards, the tail tip was cut off (1 mm), and blood was taken for glucose and insulin testing. After the whole blood had been allowed to coagulate, the supernatant was extracted. Twenty microlitres of serum per sample was required based on the blood glucose testing kit (Mlbio, Shanghai, China). After completing the sample preparation, the absorbance value was read using a microplate reader at a wavelength of 505 nm. The minimum detection limit for blood glucose was 1 nmol/mL. Blood insulin was tested using a double antibody sandwich method with an ELISA kit (Mlbio). Each sample required 10 μL of serum. Finally, the absorbance value was read at a wavelength of 405 nm. The minimum detection limit for blood insulin was 0.1 mU/L. The Bennett ISI was calculated as follows: 1/[log fasting blood glucose (mmol/L) * log fasting blood insulin (mU/L)].

### ITT

Mice were fasted overnight and intraperitoneally injected with 0.75 U/kg insulin the next morning. Before 15, 30, 45, 60, 75, 90, 105, and 120 min after injection, tail vein blood was taken to measure glucose levels. Blood glucose change curves were drawn.

### Hyperinsulinaemic–euglycaemic clamp experiment

After intraperitoneal injection of pentobarbital sodium (25 mg/kg), the right external jugular veins of mice were cannulated. After 1 week of recovery, the hyperinsulinaemic–euglycaemic clamp experiment began. After fasting overnight, 60 mU/kg insulin was administered, after which insulin was infused at a rate of 2.5 mU/kg/min. Blood glucose levels were measured every 10 min. In the meantime, 20% glucose was infused, during which the infusion rate was continuously adjusted to restore blood glucose to the baseline level. The GIR was recorded as blood glucose was in steady-state.

### Western blot analysis

Cells and tissues were obtained and placed on ice in radioimmunoprecipitation assay lysis buffer containing protease inhibitors (Boster, Shanghai, China). A protein quantitative kit (Boster) was used to quantify the total protein concentrations. Fifty micrograms of protein from each sample were subjected to 10% sodium dodecyl sulfate‒polyacrylamide gel electrophoresis and transferred to a polyvinylidene fluoride membrane (Millipore, Billerica, Massachusetts, USA) for 60 min at 100 V. Five percent nonfat milk was used to block the membrane for 1 h, and then the membrane was incubated with anti-INSR (rabbit anti-human, 1:1 000, Affinity Biosciences, Liyang, Jiangsu, China), anti-INSR-α subunit (rabbit anti-human, 1:1 000, Abcam) and anti-GAPDH (rabbit anti-human, 1:2 000, Servicebio) primary antibodies overnight at 4 °C. After washing, the membrane was incubated at room temperature with a sheep anti-rabbit secondary antibody (1:50 000, Abbkine, Wuhan, Hubei, China) conjugated with horseradish peroxidase for 2 h. An enhanced chemiluminescence kit (Thermo Fisher Scientific) was used to observe the bands.

### Haematoxylin–eosin (HE) staining

Tissues and cells were fixed with 4% paraformaldehyde for 1 h and then dehydrated with gradient concentrations (70%–100%) of alcohol. Paraffin sections were dewaxed with xylene for 30 min twice. Cell slides and tissue paraffin sections were treated with haematoxylin solution (Servicebio) for 1 min. After the samples were washed with running water, eosin solution (Servicebio) was used for staining for 2 min. An inverted microscope (Nikon, Tokyo, Japan) was used to observe the images.

### Immunofluorescence staining

After PBS washes and shaking 3 times to remove the suspended *P. gingivalis* and gingipain, the cell and tissue samples were fixed with 4% paraformaldehyde for 1 h. The cell slides and tissue paraffin sections (5 µm) were dewaxed and hydrated according to previously described processes in HE staining. Afterwards, 3% bovine serum albumin (Servicebio) was used to block slides for 30 min, and then the slides were incubated with primary antibodies at 4 °C overnight. The primary antibodies used were as follows: rabbit anti-human INSR (1:250, Affinity Biosciences) and mouse anti-human gingipain (5 µg/ml, Developmental Studies Hybrid Bank, Zurich, Swiss). Afterwards, the slides were incubated with the secondary antibodies in the dark for 50 min. The secondary antibodies were as follows: CY3-labelled goat anti-rabbit IgG (1:300, Servicebio) and Alexa Fluor 488-labelled goat anti-mouse IgG (1:400, Servicebio). Finally, the slides were stained with 1 µg/ml DAPI for 10 min. The fluorescence signals were observed by laser confocal microscopy (Leica, Wetzlar, Hessian, Germany). Nuclei in the DAPI channel were blue, positive signals in the Alexa Fluor 488 channel were green, and positive signals in the CY3 channel were red.

### Real-time quantitative PCR

Real-time quantitative PCR was carried out according to the processes described in previous studies.^47^ Total RNA (50 ng/μL) was extracted from cells and tissues using TRIzol reagent (Takara, Daliang, Liaoning, China) according to the manufacturer’s instructions. Afterwards, the total RNA was reverse-transcribed into cDNA using a HiScript II Reverse Transcriptase kit (Vazyme, Nanjing, Jiangsu, China). Real-time quantitative PCR was conducted using a SYBR green PCR kit (Takara). *GAPDH* was used as an internal reference gene to standardize the results. The PCR system was 10 μL, with 0.5 μL of DNA template and 0.5 μL of each primer. The PCR process was as follows: polymerase activation at 50 °C for 2 min; predenaturation at 95 °C for 10 min; and 45 cycles of 95 °C 15 s for denaturation and 60 °C 1 min for primer polymerization and DNA synthesis. The forward primer of *INSR* was CGCTGAGAATAACCCTGGTC; the reverse primer of *INSR* was GCTGCCATCTGGATCATTTC; the forward primer of *INSR-A* was TTTTCGTCCCCAGGCCATC; the reverse primer of *INSR-A* was GTCACATTCCCAACATCGCC; the forward primer of *GAPDH* was CAAAATGGTGAAGGTCGGTGTG; and the reverse primer of *GAPDH* was TGATGTTAGTGGGGTCTCGCTC.

### Molecular docking analysis

The protein structures of RgpB (1CRV) and INSR (2HR7) were obtained from the Protein Data Bank. The protein structures of RgpA and Kgp were calculated using the AlphaFold 2 prediction model. Water molecules and heteroatoms were deleted from the structures, and hydrogen atoms were added using the UCSF Chimera visualization system. Finally, Amber99SB charges were allocated, and protonation states were also allocated using the H++3.0 program.^48^ AutoDock 4.2 software (Olson Laboratory, San Diego, California, USA) was used for molecular docking analysis. Molecular docking was performed in flexible docking mode, with INSR as a receptor and Kgp, RgpA, and RgpB as ligands. INSR was taken as the docking centre, the box size was set to a cube with a side length of 120 Å, and the spacing step size was set to 0.375. The maximum limit for searching conformations was set to 10 000, and the Lamarckian genetic algorithm was used for conformational sampling with 100 conformation outputs per docking. After scoring, the top ten conformations were compared. The open-source software PyMOL (DeLano Scientific LLC, Gilbert, Arizona, USA) and Maestro Academic Edition (Schrödinger LLC, New York, New York, USA) were used for protein interaction analysis.

### LC–MS/MS analysis

Two micrograms of INSR α subunit (MedChemExpress, Shanghai, China) were incubated with 10 nM gingipain (Kgp or RgpA/B, Creative Enzymes, Shirley, NY, USA) at 37 °C for 30 min. Then, 1D Nano LC-MS/MS (Thermo Fisher Scientific, LC: U3000, MS: Q Exactive) analysis was performed. The sample was washed twice with 0.1% formic acid (Thermo Fisher Scientific), and 70% acetonitrile (Merck, Darmstadt, Hessian, Germany) was added. Then, the sample was centrifuged, vacuum-dried into a powder state, and dissolved with 2% acetonitrile/0.1% formic acid. The peptides were separated over 70 min at 600 nL/min. The MS settings were as follows: mass/charge ratio range, 350 to 1500; resolution for MS^1^ scan, 70 000 full width at half maximum; resolution for MS^2^ scan, 17 500 full width at half maximum; isolation window, 2.0 m/z; nce, 27; underfill ratio, 1%; dynamic exclusion, 20 s. Thermo Proteome Discoverer version 2.3 (Thermo Fisher Scientific) was used to process the raw data, and the UniProt database was used to analyse the results. The false discovery rate was < 0.1% at the peptide level.

### Modified ELISA

The INSR α subunit (0.1 mg/mL) was dissolved in pH 7.4 PBS (Thermo Fisher Scientific). Then, 100 μL of INSR protein was added to each well in high-binding ELISA plates (Costar, New York, New York, USA) for 1 h of incubation. Afterwards, the plates were cleaned 3 times with 0.1% Tween 20 (Sigma). Then, 200 μL of 2% polyvinyl alcohol was dissolved in PBS and added to each well, and the plates were incubated for 1 h. After 3 washes, 0.83 µg of insulin connected with horseradish peroxidase (Qiyuebio, Xi’an, Shaanxi, China) was dissolved in 1.0 ml of PBS, and serial dilutions were added to each well. The plates were incubated for 1 h. Finally, 100 μL of stock ABTS (2, 20-azino-bis [3-ethylbenzothiazoline-6-sulfonic acid]) (Millipore, Burlington, MA, USA) was added to each well, and the plates were incubated for 30 min. The plates were then read on a SpectraMax Plus scanning microplate spectrophotometer at 405 nm. The control well in each plate was not coated with INSR, and its reading was subtracted from the absorbance value of the experimental wells as the value for nonspecific binding. Each group had at least 3 wells, and binding curves were drawn based on the absorbance value.

### Statistical analysis

The data were analysed using SPSS version 24.0 (IBM, Armonk, New York, USA). The Kolmogorov-Smirnov test was performed to determine whether the data were normally distributed. Continuous variables with a normal distribution are presented as the mean ± standard deviation. Two groups of data were compared using Student’s *t*-test, while data from three or more groups were analysed using one-way analysis of variance and Dunnett’s multiple comparison test. The categorical variables are presented as frequencies and percentages, and the chi-squared test or Fisher’s exact test was used to analyse the differences among groups. When multiple logistic regression was used to determine the association between risk factors and IR, other factors that might affect the occurrence of IR were controlled for as confounding factors. To prevent the exclusion of possible confounding factors, *P* < 0.1 was considered to indicate statistical significance when screening for factors that might affect the development of IR. The correlation between the HOMA-IR score and the amount of isolated salivary *P. gingivalis* was evaluated using Pearson’s correlation coefficient, and a 95% confidence interval of the correlation coefficient was calculated. All statistical analyses were two-tailed, with a *P* value less than 0.05 being considered to indicate statistical significance.

### Supplementary information

Supplemental Material
