# Supplementary material for: Gingipain from Porphyromonas gingivalis causes insulin resistance by degrading insulin receptors through direct proteolytic effects
Source: Int J Oral Sci. 2024 Aug 1;16:53. doi: 10.1038/s41368-024-00313-z (PMC11291925; doi:10.1038/s41368-024-00313-z)
Supplement: Supplementary file 1 — Supplemental Material [file 41368_2024_313_MOESM1_ESM.pdf]

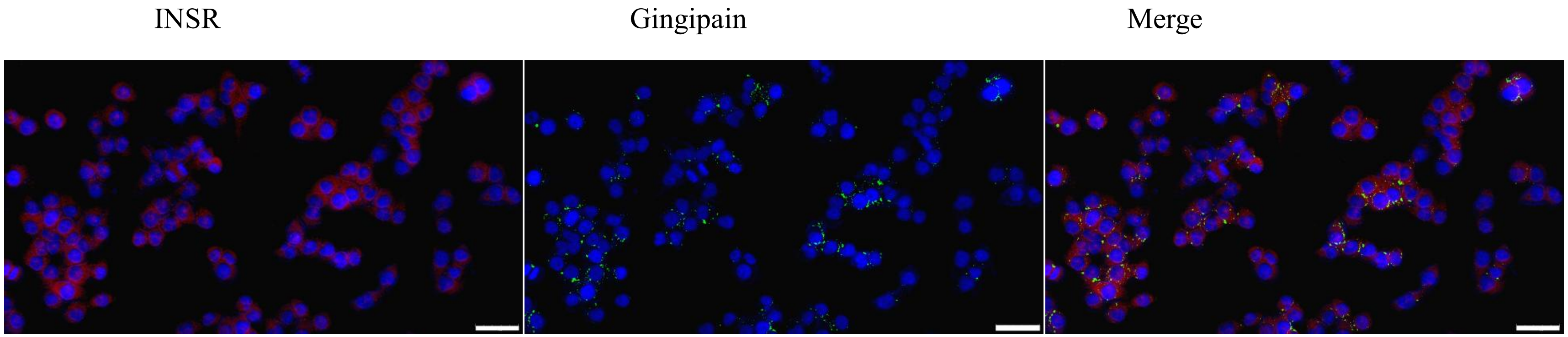

Supplementary Fig. 1 Gingipain derived from *P. gingivalis* may degrade INSR in hepatocytes

Immunofluorescence staining was used to detect INSR protein expression changes (red) in hepatocytes after coculture with *P. gingivalis* and the distribution of gingipain (green) (scale bar: 50 μm). *P. gingivalis*, *Porphyromonas gingivalis*; INSR, insulin receptor

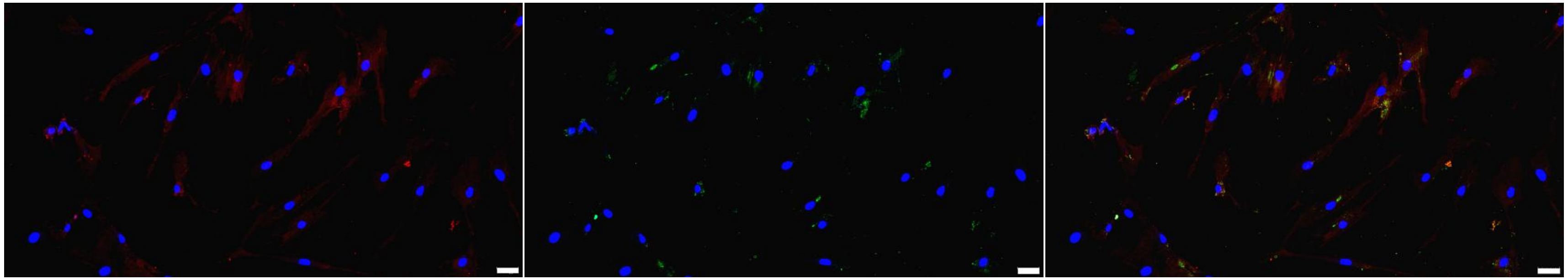

Supplementary Fig. 2 Gingipain derived from *P. gingivalis* may degrade INSR in myocytes

Immunofluorescence staining was used to detect INSR protein expression changes (red) in myocytes after coculture with *P. gingivalis* and the distribution of gingipain (green) (scale bar: 50 μm). *P. gingivalis*, *Porphyromonas gingivalis*; INSR, insulin receptor

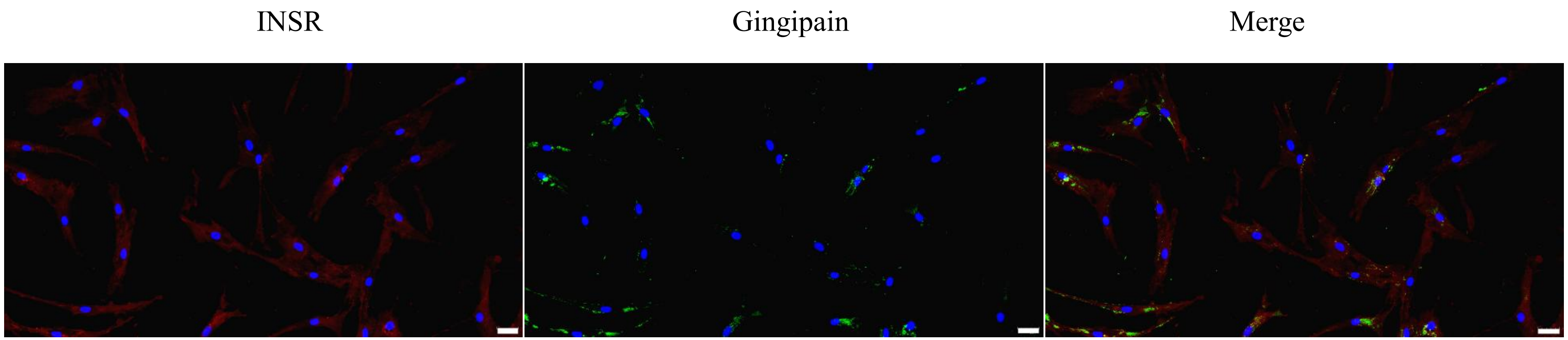

Supplementary Fig. 3 Gingipain derived from *P. gingivalis* may degrade INSR in adipocytes

Immunofluorescence staining was used to detect INSR protein expression changes (red) in adipocytes after coculture with *P. gingivalis* and the distribution of gingipain (green) (scale bar: 50 μm). *P. gingivalis*, *Porphyromonas gingivalis*; INSR, insulin receptor

INSR Gingipain Merge

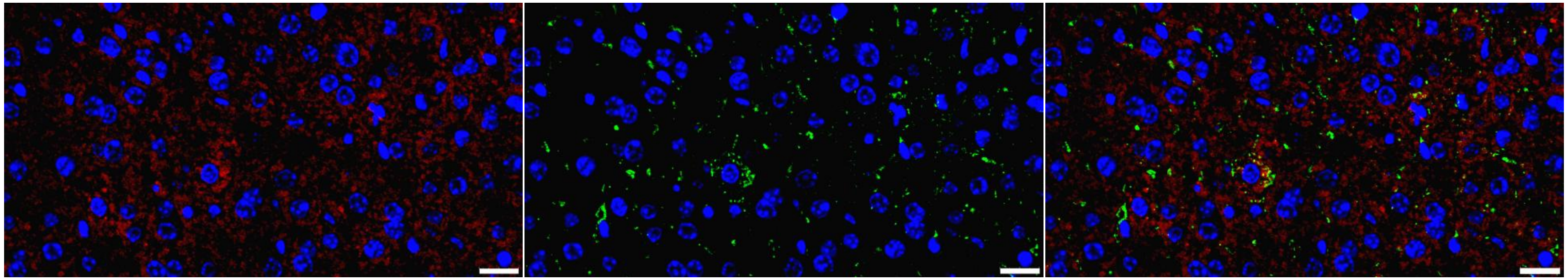

Supplementary Fig. 4 Gingipain derived from *P. gingivalis* may degrade INSR in liver

Immunofluorescence staining was used to detect the INSR protein expression changes (red) in liver of mice and the distribution of gingipain (green) (scale bar: 20 um). *P. gingivalis*, *Porphyromonas gingivalis*; INSR, insulin receptor

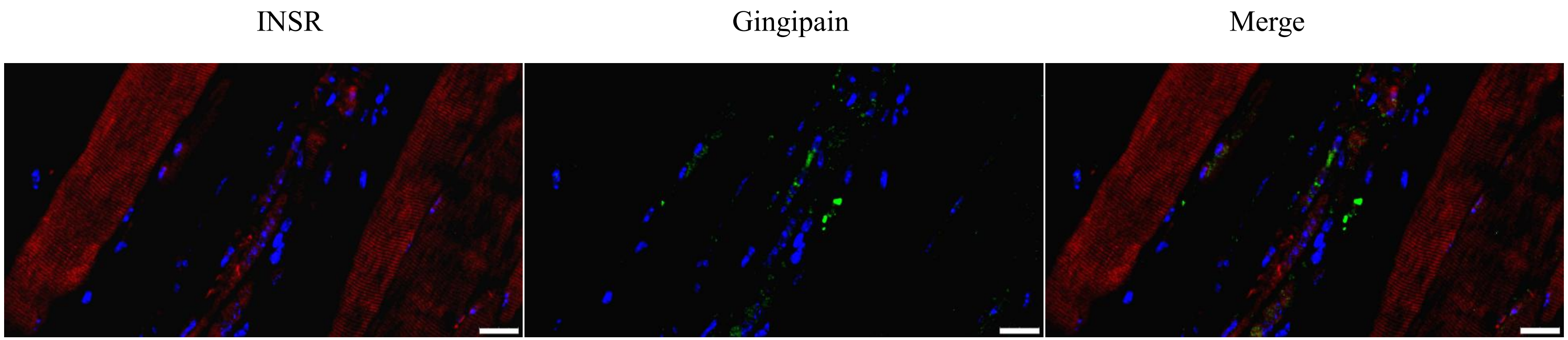

Supplementary Fig. 5 Gingipain derived from *P. gingivalis* may degrade INSR in skeletal muscle

Immunofluorescence staining was used to detect the INSR protein expression changes (red) in skeletal muscle of mice and the distribution of gingipain (green) (scale bar: 20 um). *P. gingivalis*, *Porphyromonas gingivalis*; INSR, insulin receptor

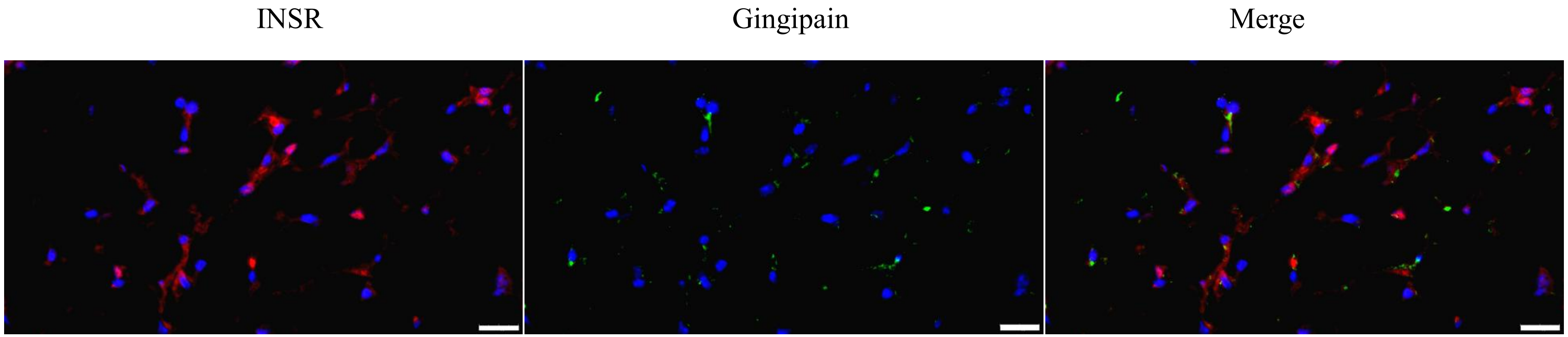

Supplementary Fig. 6 Gingipain derived from *P. gingivalis* may degrade INSR in adipose

Immunofluorescence staining was used to detect the INSR protein expression changes (red) in adipose of mice and the distribution of gingipain (green) (scale bar: 20 um). *P. gingivalis*, *Porphyromonas gingivalis*; INSR, insulin receptor

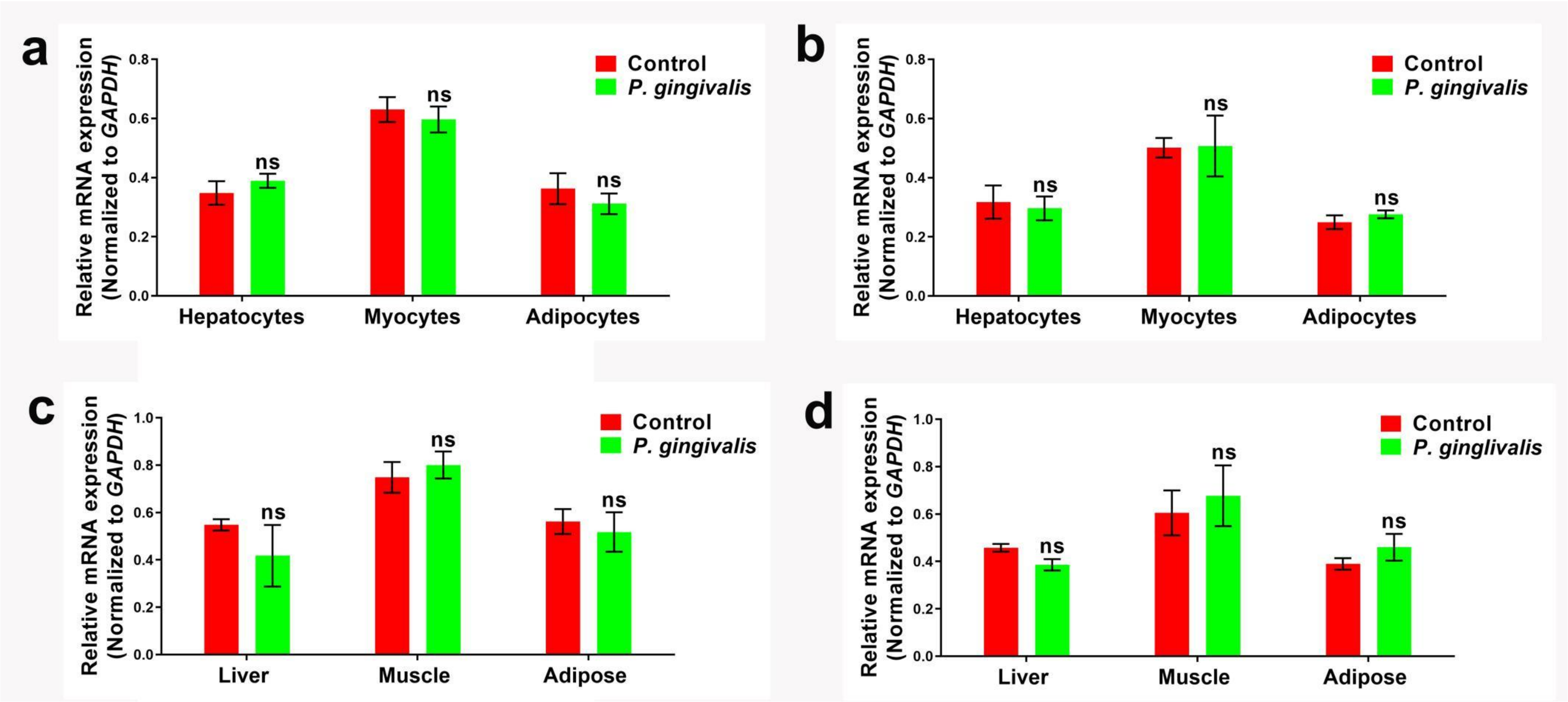

Supplementary Fig. 7 Gingipain would not affect the mRNA expression of INSR

a, b Changes in the mRNA expression of INSR (**a**) and INSR-A (**b**) in hepatocytes, myocytes and adipocytes after coculture with *P. gingivalis*. **c,d** Changes in the mRNA expression of INSR (**c**) and INSR-A (**d**) in liver, skeletal muscle and adipose tissues of mice after oral fed with *P. gingivalis*.
